# A feasibility study of sexual organ‐dose sparing volumetric modulated arc therapy in female low rectal cancer patients treated with three‐dimensional conformal radiation therapy

**DOI:** 10.1002/acm2.70394

**Published:** 2025-11-27

**Authors:** Margaret H. Downes, Victoria Olsen, Andre Williams, Rendi Sheu, Maria Thor, Lucy Greenwald, Vishruta Dumane, Kristin Hsieh, Ayesha Ali, Orly Morgan, Michael Buckstein, Deborah C. Marshall

**Affiliations:** ^1^ Icahn School of Medicine at Mount Sinai New York New York USA; ^2^ Department of Radiation Oncology Icahn School of Medicine at Mount Sinai New York New York USA; ^3^ Department of Population Health Science and Policy Icahn School of Medicine at Mount Sinai New York New York USA; ^4^ Division of Anatomy Department of Surgery University of Toronto Toronto Ontario Canada; ^5^ Department of Medical Physics Memorial Sloan Kettering Cancer Center New York New York USA; ^6^ Department of Radiation Oncology Thomas Jefferson University Hospitals Philadelphia Pennsylvania USA; ^7^ Miller School of Medicine at University of Miami Miami Florida USA

**Keywords:** 3D‐CRT, dosimetric analysis, IMRT, photon, radiation treatment, rectal cancer, sexual organs at risk

## Abstract

**Background:**

Sexual dysfunction is a significant toxicity of pelvic radiation therapy (RT) for female rectal cancer patients and may be more common with intensity‐modulated techniques compared to three‐dimensional conformal radiation therapy (3D‐CRT). However, limited research has evaluated dose sparing of female sexual organs at risk (OARs), particularly erectile tissues that are critical for sexual function.

**Purpose:**

This feasibility study aimed to demonstrate the practicality of contouring and sparing female sexual OARs in rectal cancer RT by generating sexual organ–sparing volumetric modulated arc therapy (SO‐VMAT) plans from a small 3D‐CRT cohort and comparing dose distributions to guide future planning.

**Methods:**

Nine female patients with low rectal adenocarcinoma treated with concurrent chemoradiation using 3D‐CRT were retrospectively analyzed. Sexual OARs (bulboclitoris, external genitalia, and vagina) were contoured and incorporated into replanned SO‐VMAT plans. Institutional dose–volume constraints were applied for standard OARs and planning target volumes (PTVs). SO‐VMAT and 3D‐CRT plans were normalized to equal PTV coverage, and dose–volume metrics were compared using Wilcoxon signed‐rank tests.

**Results:**

Contouring and sparing of female sexual OARs was feasible in all cases. Compared to 3D‐CRT, SO‐VMAT reduced radiation doses to sexual OARs. For the bulboclitoris, SO‐VMAT lowered V3000cGy and V4000cGy (both *p* = 0.02). For the external genitalia, SO‐VMAT reduced maximum and mean dose (both *p* = 0.02) as well as V1000cGy (*p* = 0.004) and V2000cGy (*p* = 0.01). Vaginal sparing was observed at V4000cGy (*p* = 0.03). SO‐VMAT provided significant bladder sparing, whereas bowel and femoral head doses remained comparable. Target coverage remained equivalent between techniques.

**Conclusion:**

This study demonstrates that female sexual OARs can be systematically contoured and effectively spared during rectal cancer RT using SO‐VMAT. Compared with 3D‐CRT, SO‐VMAT achieved meaningful dose reductions while maintaining target coverage. These preliminary findings support multi‐institutional studies with patient‐reported outcomes to validate clinical relevance and guide integration of female sexual OARs into treatment planning.

## 1 INTRODUCTION

Rectal cancer is the third most prevalent and second most deadly cancer in the United States, with a rising global incidence.^1^, ^2^ In recent decades, the proportion of rectal cancer cases in individuals younger than 55 years has increased at an alarming rate.^3^, ^4^ As the incidence of rectal cancer continues to rise, particularly in younger populations, and advancements in cancer diagnostics and therapies improve survival, the number of long‐term rectal cancer survivors is also growing.^4^, ^5^ These trends underscore the increasing importance of addressing the long‐term toxicities of curative treatments and optimizing posttreatment quality of life, given the potential for significant functional and psychosocial impacts following treatment.

Pelvic radiotherapy (RT) puts patients at risk for long‐term radiation toxicities affecting various pelvic tissues, including bones, vessels, nerves, and the gastrointestinal (GI), genitourinary (GU), and gynecological systems. Sexual dysfunction is a distressing and underrecognized side effect of RT for patients with rectal cancer, significantly impacting quality of life.^6^, ^7^ While the sexual side effects of pelvic RT have been studied extensively in male patients,^8^ sexual toxicities in females remain underexplored, with most research focusing on vaginal changes and overlooking other key sexual structures. One such structure—the bulboclitoris, comprised of the clitoris and vestibular bulbs—plays a critical role in genital arousal and orgasm but is rarely considered during RT planning.^9^

In addition, while the vagina and external genitalia are sometimes included in treatment fields, they are rarely contoured systematically or with standardized detail, limiting their protection during planning. Given the close proximity of these sexual OARs to the rectum and the rich vascular, neural, and mucosal composition of these tissues, female sexual OARs are likely highly sensitive to radiation exposure, which may disrupt both structural integrity and function. As a result, these organs may be particularly susceptible to RT‐induced dysfunction. Addressing the current lack of systematic contouring and dose–volume data for female sexual OARs requires demonstrating the feasibility of delineating these structures and establishing preliminary benchmarks for achievable sparing.

Evidence from clinical trials underscores the importance of the need to better understand RT‐related sexual toxicities related to different treatment approaches. In an effort to understand toxicities related to different treatment approaches, chemotherapy alone was compared to chemoradiotherapy in the PROSPECT trial, and female patients in the chemoradiotherapy arm experienced greater diminished sexual function as compared to chemotherapy alone, highlighting the need for strategies to minimize RT‐related sexual toxicity.^10^ As newer approaches such as total neoadjuvant therapy (TNT) and nonoperative management evolve, reducing radiation‐induced sexual dysfunction remains a critical area for innovation. Established by trials such as RAPIDO and PRODIGE‐23,^11^, ^12^ TNT has become a standard approach for patients with locally advanced rectal cancer by utilizing upfront pelvic RT and systemic treatment.^13^

Advances in RT techniques have reduced radiation‐related toxicities by enabling more precise tumor targeting while minimizing radiation exposure to surrounding normal tissues.^14^, ^15^ For many cancers, intensity‐modulated radiation therapy (IMRT) and volumetric modulated arc therapy (VMAT) have been shown to decrease RT‐related toxicities compared to three‐dimensional conformal radiation therapy (3D‐CRT).^16^, ^17^, ^18^ However, for patients with rectal cancer, 3D‐CRT remains a guideline‐recommended technique since randomized trials have not demonstrated significant reductions in gastrointestinal (GI) toxicity with IMRT or VMAT.^19^ Notably, these trials have focused on GI outcomes and largely overlooked other toxicities, such as genitourinary and sexual dysfunction. In PROSPECT, 51% of patients received 3D‐CRT and 49% received IMRT, yet neither group incorporated standardized sparing of female erectile tissues in treatment planning.^10^ More recently, an analysis of the RAPIDO trial reported that IMRT was associated with higher rates of late sexual toxicity compared to 3D‐CRT despite no overall differences in GI or GU outcomes.^20^ This finding highlights a critical limitation: simply adopting advanced RT techniques does not guarantee reduced toxicity without deliberate efforts to contour and spare relevant OARs. These results underscore the need for intentional sexual organ–sparing VMAT (SO‐VMAT) approaches that specifically account for female sexual OARs in treatment planning.

In this study, we aim to understand the distribution of dose to the sexual OARs during standard 3D‐CRT and compare SO‐VMAT with 3D‐CRT to evaluate the feasibility and dose–volume benefits of introducing dose–volume histogram (DVH) constraints to female sexual structures during RT for rectal cancer.

## 2 METHODS

### 2.1 Patients

Nine consecutive patients with histologically confirmed invasive adenocarcinoma of the low rectum, where the planning target volume (PTV) extended caudally to the ventral aspect of the pubic symphysis, treated with curative chemoradiation using the 3D‐CRT technique between 2017 and 2020 were included. All cases were treated by a single physician at the same clinical site to ensure consistency in simulation, contouring, and treatment planning for this proof‐of‐concept feasibility analysis. Patients who received palliative treatment or underwent primary surgical treatment were excluded. All procedures adhered to standard clinical practices, including digital examination, rectoscopy, and magnetic resonance imaging (MRI), or computed tomography (CT) imaging of the abdomen and pelvis.

### 2.2 Patient setup and CT simulation

CT simulation scans with a 3‐mm slice thickness were obtained for each patient using a Philips Big Bore CT scanner. During simulation, patients were positioned in either the prone or supine position, as per the physician preference, with an immobilization device. Vaginal dilators were not utilized as part of standard practice in this cohort. Contouring was performed on the planning CT scans, which were fused with pelvic MRI for enhanced anatomical detail. High‐resolution axial T2‐weighted sequences (1 mm slices) were most useful for visualization of erectile tissues, with T1 post‐contrast sequences incorporated when available to aid anatomical confirmation.

### 2.3 Standard OAR contours

The standard nonsexual organs at risk (OARs) were contoured as part of routine clinical practice and included the small and large bowel (extending 2 cm superior to the target volume), bladder, femoral heads/necks, iliac crests, and skin (3 mm in depth from the external body).^21^, ^22^

### 2.4 Sexual OAR contours

**FIGURE 1:**
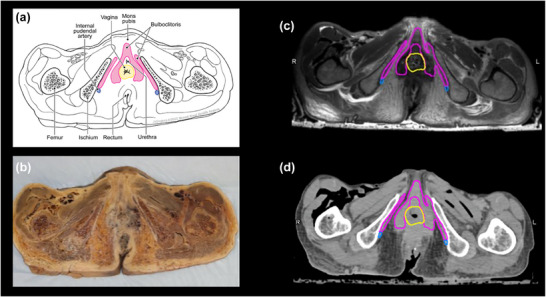
Multimodal imaging of the bulboclitoris and surrounding structures. (a) Anatomical drawing illustrating the bulboclitoris (pink) and adjacent structures, including the vaginal wall (yellow) and internal pudendal artery (blue). (b) Gross frozen cross‐sectional slice from a donated cadaver pelvis. (c) Magnetic resonance image (MRI) obtained using a Siemens T2‐weighted 3‐Tesla Skyra scanner in 1‐mm axial slices. (d) Simulation computed tomography (CT) scan of the same axial slice, performed on the Philips Brilliance Big Bore scanner in 1‐mm axial slices. These images highlight the anatomical relationships and visualization of the bulboclitoris across different imaging modalities, facilitating precise delineation for radiation treatment planning.

Sexual OARs (including the external genitalia, vagina, and bulboclitoris) were contoured for SO‐VMAT re‐planning. Contouring was performed by an anatomist to ensure anatomical precision and subsequently reviewed and approved by a board‐certified radiation oncologist with expertise in genito–pelvic anatomy. The external genitalia were contoured per standard guidelines, extending from the vaginal introitus anteriorly to the pubic symphysis and encompassing the labial skin, subcutaneous fat, and external portion of the clitoral glans. Clinical contours of some external genital structures were included as part of the original treatment plans per physician discretion; however, for consistency and accuracy, all genital structures were systematically recontoured based on a standardized anatomical atlas for SO‐VMAT planning.^19^ The bulboclitoris includes the paired vestibular bulbs (corpora spongiosa), clitoral body, and crura (corpora cavernosa; Figure 1).^23^ It was delineated beginning at the level of the urethral meatus and extending cranially along the clitoral shaft and caudally through the vestibular bulbs, which were best visualized on T2‐weighted MRI adjacent to the anterior vaginal wall and pubic symphysis. The vaginal canal was contoured from the introitus to the inferior cervical os, with MRI aiding in distinguishing the vaginal walls from adjacent bladder and rectum.

### 2.5 Target contours

For standard 3D‐CRT, the target volume included the mesorectum, presacral and internal iliac nodes as well as a 2 cm margin superior–inferior to the GTV. The multileaf collimators (MLCs) were set at the discretion of the treating physician. For the SO‐VMAT plans, PTVs were generated to mirror the treated volume targeted in original 3D‐CRT plans using the standard clinical target volumes (CTVs) that encompass the mesorectum, presacral and internal iliac nodes, and a 2 cm margin superior–inferior to the GTV, with an additional 7 mm margin for the PTV.^24^ Both 3D‐CRT and VMAT utilized a boost volume, when applicable, targeting the mesorectum and presacral nodes 2 cm superior/inferior to the GTV.

**FIGURE 2:**
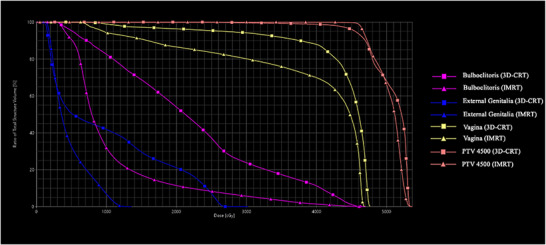
Dose‐volume histograms (DVHs) comparing 3D‐CRT and SO‐VMAT plans. Dose–volume histograms for critical structures (bulboclitoris, external genitalia, and vagina) and the planning target volume (PTV 4500 cGy) in 3D conformal radiation therapy (3D‐CRT) versus sexual organ sparing intensity‐modulated radiation therapy (SO‐VMAT). The curves illustrate the percentage of each structure receiving a given dose of radiation (in cGy). SO‐VMAT demonstrates improved sparing of critical structures, including the bulboclitoris and external genitalia, while maintaining comparable PTV coverage, as indicated by the overlapping PTV 4500 curves.

**FIGURE 3:**
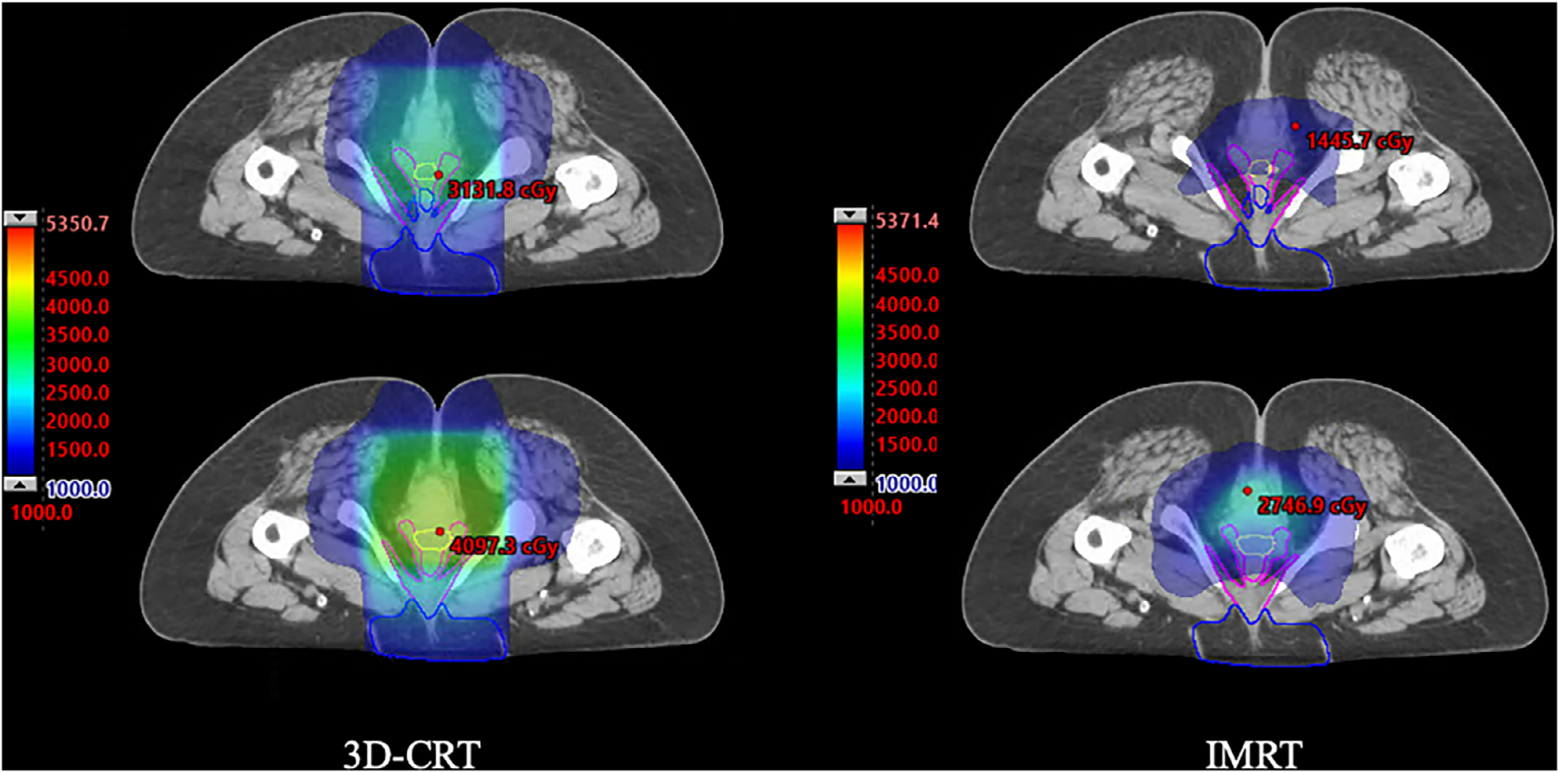
Dose distribution comparison between 3D‐CRT and SO‐VMAT plans. Representative axial CT images illustrating radiation dose distribution for 3D conformal radiation therapy (3D‐CRT, left) and sexual organ sparing intensity‐modulated radiation therapy (SO‐VMAT, right). The color wash represents dose gradients in centigray (cGy), with higher doses shown in red and lower doses in blue. Mean dose to the bulboclitoris is indicated for each technique, demonstrating significantly lower radiation exposure with IMRT (right panels). SO‐VMAT achieved improved sparing of surrounding critical structures while maintaining adequate target coverage compared to 3D‐CRT.

### 2.6 Treatment planning

#### 2.6.1 3D‐CRT

The 16‐MV 3D‐CRT plans were generated in routine clinical practice using the Eclipse treatment planning system (Varian Medical Systems, Palo Alto, CA; Version 15, anisotropic analytical algorithm) with standard prescription objectives and OAR constraints. The prescription dose was 50.4 Gy delivered in 28 fractions, with a sequential boost applied when indicated. Planning objectives included equal or greater than 95% coverage of the PTV with the prescription dose, a maximum dose constrained to remain below 110% of the prescription dose, and minimization of OAR doses according to the as‐low‐as‐reasonably‐achievable (ALARA) principle. Typical institutional OAR constraints included small and large bowel D200cc less than 4500 cGy, bladder V5000cGy less than 50%, and femoral heads V3000cGy less than 50%. All clinical treatment plans were reviewed and approved by the treating board‐certified radiation oncologist.

#### 2.6.2 SO‐VMAT

All VMAT plans in this study were generated using 6 MV volumetric modulated arc therapy with the Eclipse treatment planning system, using three arcs for the initial plan and two arcs for the boost plan. Rotational IMRT, or VMAT, was selected over static‐field IMRT for this analysis, as VMAT allows for more efficient modulation around small, irregular targets and reduces beam‐on time. All SO‐VMAT plans were normalized to have similar PTV coverage to corresponding target coverage of 3D‐CRT plans, standard nonsexual organ sparing to meet corresponding 3D‐CRT constraints, and sexual OARs were spared ALARA. The maximum dose was kept < 106% of the prescription dose in regions where the PTVs overlapped with adjacent structures. In non‐overlapping regions (e.g., bulboclitoris‐PTV interface), we cropped the OARs by a small margin, applied dose–volume objectives in Eclipse, and manually iterated constraint values and priorities to shift high‐ and intermediate‐dose regions away from sexual structures without compromising PTV coverage.

### 2.7 Analysis

Dose–volume histograms (DVHs) were generated for OARs, and DVH metrics were compared between the 3D‐CRT and SO‐VMAT plans. Wilcoxon signed‐rank tests were used to compare dose metrics for each OAR between the two techniques. In addition, isodose line (IDL) volumes at 5%, 50%, 100%, and 105% of the prescription dose were extracted from each plan and compared between 3D‐CRT and SO‐VMAT to facilitate evaluation of dose coverage and distribution differences between techniques. Relative change calculations were performed to assess differences in dose distribution patterns across techniques. A two‐sided p‐value with an *α* level of 0.05 was considered statistically significant for all analyses. Statistical analyses were performed using RStudio Version 4.4.1.

## 3 RESULTS

Demographic and clinical characteristics of the nine included patients are summarized in Table 1. The median age of the nine participants was 59 years (interquartile range [IQR]: 48–64). Each patient received a median radiation dose of 5040 centigray (cGy) (IQR: 5040–5040). Among the patients, 33% (*n* = 3) had Stage IIA disease, while 67% (*n* = 6) had Stage III disease, including five with Stage IIIB and one with Stage IIIC. The median distance of the tumor from the anal verge was 4.4 cm (IQR: 4.1–7.7). Volumetric data for the bulboclitoris, external genitalia, and vagina are summarized in Table 1.

**TABLE 1:** Characteristics of patients with low rectal adenocarcinoma treated with standard 3D‐CRT.

Characteristic	Value (*N* = 9) Median (IQR) or *n* (%)
Age, years	59 (48–64)
Stage	
IIA	3 (33%)
IIIB	5 (55%)
IIIC	1 (11%)
Radiation dose, cGy	5040 (5040–5040)
Concurrent chemotherapy, *n* (%)	9 (100%)
Distance of tumor from anal verge, cm	4.4 (4.1–7.7)
Bulboclitoris volume, cc	17.3 (14.4–21.1)
Bulboclitoris‐PTV volume, cc	16.4 (14.2–21.0)
External genitalia volume, cc	123.5 (100.9–197.9)
Vagina volume, cc	7.6 (7.0–8.2)

Abbreviations: 3D‐CRT: Three‐dimensional conformal radiation therapy; cGy: centigray; IQR: interquartile range; PTV: planning target volume.

Systematic reductions in radiation doses to sexual OARs was observed with SO‐VMAT compared to 3D‐CRT (Table 2): For the bulboclitoris, SO‐VMAT reduced the median D2cc dose from 4105.2 cGy (IQR: 2710.9–4415.1) to 2965.0 cGy (IQR: 1528.7–4476.9; *p* = 0.20) and the D50% dose from 2118.8 cGy (IQR: 739.5–2444.7) to 1163.0 cGy (IQR: 616.3–1530.1; *p* = 0.07) but did not reach statistical significance. The other investigated sexual OAR DVH metrics, such as V1000cGy, V2000cGy, V3000cGy, and V4000cGy, were all statistically significantly reduced using SO‐VMAT (all *p* ≤ 0.02). When excluding the overlap between the bulboclitoris and the PTV (PTV 4500), the bulboclitoris‐PTV exhibited similar reductions in high‐dose exposure, with significantly lower V1000cGy (*p* = 0.01), V2000cGy (*p* = 0.01), V3000cGy (*p* = 0.02), and V4000cGy (*p* = 0.04), while metrics such as D2cc, median, and the mean dose were numerically, though not statistically significantly, lower.

**TABLE 2:** Dosimetric comparison of standard 3D‐CRT and SO‐VMAT plans for sexual organs at risk in low rectal cancer patients.

Structure	Structure metric	3D‐CRT median (IQR)	SO‐VMAT median (IQR)	Relative change Median (IQR)	*p*‐value
**Bulboclitoris**					
	D2cc, cGy	4105.2 (2710.9–4415.1)	2965.0 (1528.7–4476.9)	−22.9% (‐45.2% to ‐1.8%)	0.20
	D50%, cGy	2118.8 (739.5–2444.7)	1163.0 (616.3–1530.1)	−27.8% (‐62.8% to ‐18.6%)	0.07
	Maximum Dose, cGy	4626.9 (2893.5–4859.1)	4790.1 (4699.0– 4855.5)	1.6% (‐0.1% to 5.4%)	0.36
	Mean Dose, cGy	2090.5 (1076.5–2571.6)	1751.3 (873.5–2153.0)	−26.4% (‐45.8% to ‐14.9%)	0.07
	V1000cGy, %	82.6 (44.7–100)	58.8 (16.1–66.7)	−37.6% (‐62.0% to ‐12.5%)	0.01*
	V2000cGy, %	54 (24.8–85.5)	33.3 (7.2–39.4)	−59.5% (‐72.9% to ‐38.2%)	0.01*
	V3000cGy, %	23.8 (18.1–51.3)	16.4 (4.5–27.3)	−58.9% (‐70.8% to ‐27.1%)	0.02*
	V4000cGy, %	11.3 (10.4–37.7)	6.4 (1.4–17.5)	−64.3% (‐82.2% to ‐44.2%)	0.02*
**Bulboclitoris—PTV**					
	D2cc, cGy	3861.8 (1116.3–4092.8)	1656.2 (1393.2–3493.8)	−34.7% (‐55.4% to ‐12.4%)	0.13
	D50%, cGy	886.2 (530.6–2113.4)	786.8 (467.8–1498.7)	−27.7% (‐55.0% to ‐18.6%)	0.10
	Maximum Dose, cGy	4505.8 (2348.4–4814.9)	4692.9 (1859.2–4795.5)	−1.0% (‐40.9% to 5.4%)	0.91
	Mean Dose, cGy	1501.5 (653.7–2187.2)	1067.2 (481.2–1736.8)	−26.4% (‐47.3% to ‐15.8%)	0.13
	V1000cGy, %	76.2 (44.3–82.9)	31.3 (9.3–66)	−51.7% (‐68.4% to ‐13%)	0.01*
	V2000cGy, %	51.6 (24.3–54.5)	10.7 (0–34.5)	−67.2% (‐85% to ‐42.8%)	0.01*
	V3000cGy, %	20.5 (17.6–36)	4.9 (0–20.6)	−59.5% (‐78.5% to ‐32.2%)	0.02*
	V4000cGy, %	10.6 (0–19.4)	1.5 (0–6.2)	−76.5% (‐85.4% to ‐52.7%)	0.04*
**External Genitalia**					
	D50%, cGy	521.5 (315.6–678.9)	357.0 (305.8–363.2)	−31.5% (‐47.1% to ‐6.1%)	0.07
	Maximum Dose, cGy	2709.8 (2492.1–3021.8)	1439.2 (1361.1–1715.6)	−46.3% (‐52.7% to ‐45.5%)	0.02*
	Mean Dose, cGy	872.5 (634.8–997.5)	461.8 (444.6–512.0)	−48.5% (‐53.7% to ‐45.6%)	0.02*
	V1000cGy, %	41.6 (25.5–44.8)	5.9 (3.5–9.3)	−83.7% (‐87.5% to ‐79.1%)	0.004**
	V2000cGy, %	18.4 (10.6–26.6)	0 (0–0)	−100% (‐100% to ‐100%)	0.01*
	V3000cGy, %	0 (0‐0)	0 (0–0)	−100% (‐100% to ‐100%)	0.37
**Vagina**					
	D50%, cGy	4564.2 (4231.4–4782.2)	4718.3 (3406.1–5038.0)	−1.3% (‐22.6% to 5.5%)	0.91
	Maximum Dose, cGy	3999.5 (3396.8–4704.4)	4218.8 (3016.4–4743.2)	−4.3% (‐16.5% to 2.7%)	0.36
	V2000cGy, %	99.0 (87.4–100)	94.8 (72.2–100)	0% (‐17.4% to 0%)	0.11
	V3000cGy, %	96.4 (78.4–100)	86.6 (59.7–97.8)	−4.8% (‐23.9% to 0%)	0.05
	V4000cGy, %	94.3 (71.4–100)	80.2 (52.8 – 96.0)	−9.4% (‐26.1% to 0%)	0.03*
	V5000cGy, %	88.0 (60.4–100)	70.3 (44.7–92.9)	−15.2% (‐25.9% to 0%)	0.15

Abbreviations: 3D‐CRT: Three‐dimensional conformal radiation therapy; D2cc: Dose received by the most exposed 2 cm^3^ of the structure in centigray; D50%: Dose received by 50% of the structure's volume in centigray; Maximum Dose: Dose received by the most exposed 0.03 cm^3^ of the structure in centigray; SO‐VMAT: Sexual organ sparing volumetric modulated arc therapy; Vx cGy: Percent volume of the structure receiving at least x centigray (cGy).

* p < 0.05.

** p < 0.01.

Similar trends were observed for the external genitalia, where SO‐VMAT significantly lowered the maximum dose (2709.8 cGy [2492.1–3021.8] vs. 1439.2 cGy [1361.1–1715.6], *p* = 0.02), mean dose (872.5 cGy [634.8–997.5] vs. 461.8 cGy [444.6–512.0], *p* = 0.02), V1000cGy (41.6% [25.5–44.8] vs. 5.9% [3.5–9.3], *p* = 0.004), and V2000cGy (18.4% [10.6–26.6] vs. 0% [0–0], *p* = 0.01). In the vagina, significant reductions were seen in V4000cGy (94.3% [71.4–100] vs. 80.2% [52.8–96], *p* = 0.03), with a similar dose reduction trend was also observed in V3000cGy (96.4% [78.4–100] vs. 86.6% [59.7–97.8], *p* = 0.05). Figure 2 further highlights these differences, as the DVHs demonstrate reduced radiation exposure to the bulboclitoris, external genitalia, and vagina with SO‐VMAT compared with 3D‐CRT.

In addition to the dose reductions observed for sexual OARs, SO‐VMAT compared to standard 3D‐CRT also demonstrated advantages for certain typically nonsexual involving structures, particularly the bladder (Table 3). SO‐VMAT significantly reduced the percent volume of the bladder receiving at least 3500 cGy (V3500cGy: 63.7% [IQR: 61–80.1] vs. 44.2% [34–46.4], *p* = 0.004) and 4500 cGy (V4500cGy: 53% [45–60] vs. 24.4% [13.8–31.7], *p* = 0.004), with a trend toward reduction in V5000cGy (29.2% [16.7–43.9] vs. 12.9% [6.5–19.3], *p* = 0.05). In contrast, no significant dose differences were observed for the bowels (both small and large) or femoral heads, where maximum doses and DVH metrics remained comparable between 3D‐CRT and SO‐VMAT. For instance, the D200cc dose to the small bowel was 2776.7 cGy (IQR: 966.0–2938.7) with 3D‐CRT and 2261.1 cGy (945.0–2291.2) with SO‐VMAT (*p* = 0.57). Similarly, the percent volume receiving at least 3000 cGy (V3000cGy) for the femoral heads showed no significant differences (2.8% [IQR: 1.7–12.0] vs. 12.4% [7.3–15.7], *p* = 1.00) between 3D‐CRT and SO‐VMAT. Regarding dose to the skin surface, SO‐VMAT significantly reduced V2000cGy (1.1% [1.1–1.8] vs. 8.5% [4.9–9.1], *p* = 0.004), while V3000cGy was very low and comparable between techniques (0% [0–0] with SO‐VMAT vs. 0.1% [0–2.2] with 3D‐CRT, *p* = 0.06). In contrast, V500cGy was higher with SO‐VMAT (52.3% [43.7–52.7] vs. 38.5% [36.8–50.8], *p* = 0.01).

**TABLE 3:** Dosimetric comparison of 3D‐CRT and SO‐VMAT plans for standard nonsexual organs at risk in rectal cancer patients.

Structure	Structure metric	3D‐CRT median (IQR)	SO‐VMAT median (IQR)	Relative change median (IQR)	*p*‐value
**Bladder**					
	Maximum dose, cGy	5306.0 (5259.3–5363.7)	5343.8 (5328–5389.7)	1.3% (0% to –3.1%)	0.04*
	V3500cGy, %	63.7 (61.0–80.1)	44.2 (34.0–46.4)	−54.9% (‐58.5% to ‐30.1%)	0.004**
	V4500cGy, %	53.0 (45.0–60.0)	24.4 (13.8–31.7)	−‐69.0% (‐75.9% to ‐44.6%)	0.004**
	V5000cGy, %	29.2 (16.7–43.9)	12.9 (6.5–19.3)	−72.4% (‐80.7% to ‐33.8%)	0.05
**Large Bowel**					
	D200cc, cGy	109.8 (67.7–578.2)	472 (188.1–970.9)	78.9% (44.9% to 307.1%)	0.08
	Maximum dose, cGy	5327.2 (5297.8–5358.4)	5339.2 (5325.1–5356.4)	0.1% (‐0.1% to 0.2%)	0.47
**Small Bowel**					
	D200cc, cGy	2602.9 (966.0–2922.1)	2261.1 (945.0–2291.2)	−2.2% (‐22.0% to 7.0%)	0.25
	Maximum dose, cGy	4903.5 (3116.5–5293.2)	5055.4 (4815.2–5318.1)	0.5% (‐3.3% to 6.6%)	0.73
**Left Femoral Head**					
	Maximum Dose, cGy	3756.3 (3232.0–4321.0)	4102.0 (3963.4–4266.5)	5.5% (‐5.1% to 8.5%)	0.50
	V3000cGy, %	2.7 (1.5–15.8)	11.3 (7.6–13.5)	279.2% (‐25.9% to 404%)	0.43
**Right Femoral Head**					
	Maximum dose, cGy	3296.7 (3258.4–4154.0)	4135.5 (3786.1–4336.5)	7.7% (‐8.9% to 30.7%)	0.36
	V3000cGy, %	2.9 (1.8–8.2)	13.5 (6.9–17.8)	201.0% (‐19.4% to 667.7%)	0.50
**Skin Surface**					
	V500cGy, %	38.5 (36.8–50.8)	52.3 (43.7–52.7)	21.6% (13.7% to 27.5%)	0.01*
	V2000cGy, %	8.5 (4.9–9.1)	1.1 (1.1–1.8)	−80.0% (‐87.9% to ‐77.8%)	0.004**
	V3000cGy, %	0.1 (0–2.2)	0 (0–0)	−100% (‐100% to 84.4%)	0.06

Abbreviations: 3D‐CRT: Three‐dimensional conformal radiation therapy; D200cc: Dose received by the most exposed 200 cm^3^ of the structure in centigray; SO‐VMAT: Sexual organ sparing volumetric modulated arc therapy; Vx cGy: Percent volume of the structure receiving at least x centigray (cGy).

* p < 0.05.

** p < 0.01.

To further compare dose distribution patterns between 3D‐CRT and SO‐VMAT, we analyzed IDL volumes at 5%, 50%, 100%, and 105% of the plan sum and 100% of the initial plan (Table 4). SO‐VMAT significantly reduced the volumes encompassed by the 100% plan sum (*p* = 0.004), 100% initial plan (*p* = 0.004), and 50% plan sum (*p* = 0.004) IDL compared to 3D‐CRT, reflecting improved high‐ and intermediate‐dose conformality. Although the 105% plan sum IDL volume was numerically lower with SO‐VMAT, this difference did not reach statistical significance (*p *= 0.10). In contrast, the 5% plan sum IDL volume was significantly larger with SO‐VMAT compared to 3D‐CRT (*p* = 0.004). In Figure 3, the dose distribution maps illustrate that SO‐VMAT provided superior sparing of the bulboclitoris, external genitalia, and vagina in addition to reducing higher IDL volumes compared to 3D‐CRT, while maintaining adequate target coverage.

**TABLE 4:** Dosimetric comparison of isodose line volumes between 3D‐CRT and SO‐VMAT plans.

Structure	3D‐CRT median (IQR)	SO‐VMAT median (IQR)	Relative change median (IQR)	*p*‐value
IDL 105% Volume (Plan Sum)	114.7 (64.8–439.1)	61.5 (49.0–91.8)	−57.6% (‐89.7% to ‐6.1%)	0.10
IDL 100% Volume (Plan Sum)	1072.3 (895.4–1227.7)	643.9 (596.8–888.0)	−23% (‐35.3% to ‐18.9%)	0.004*
IDL 100% Volume (Initial)	1814.0 (1772.0–2137.8)	1236.0 (1170.3–1313.8)	−35.6% (‐36.4% to ‐29.6%)	0.004*
IDL 50% Volume (Plan Sum)	5237.4 (4998.9–6151.3)	3347.0 (2887.0–3505.0)	−42.3% (‐45.6% to ‐39.0%)	0.004*
IDL 5% Volume (Plan Sum)	11818.7 (11186.1–12684.1)	13424.2 (11733.7–14321.0)	12.9% (4.9% to 14.7%)	0.004*

Abbreviations: 3D‐CRT: three‐dimensional conformal radiation therapy; IDL: isodose line; IQR: interquartile range; SO‐VMAT: sexual organ‐sparing intensity‐modulated radiation therapy.

## 4 DISCUSSION

This study demonstrates that SO‐VMAT significantly reduces radiation exposure to sexual OARs and the bladder compared to standard 3D‐CRT in female patients with rectal cancer, while maintaining comparable PTV coverage and radiation doses to other critical OARs, such as the bowel and femoral heads. Key dosimetric indices were significantly lower for the bulboclitoris, external genitalia, vagina with SO‐VMAT without compromising PTV coverage, ensuring therapeutic efficacy while minimizing radiation exposure to sexual OARs. Additionally, we confirmed that the bulboclitoris can be visualized and delineated on both CT and MRI, demonstrating the feasibility of contouring this structure in rectal cancer patients to minimize radiation exposure to erectile tissues during radiotherapy.

Our results highlight that SO‐VMAT offers meaningful dosimetric advantages for sexual OARs, particularly in reducing higher dose–volume exposure. Significant reductions were observed in the percent volume receiving 1000–4000 cGy (V1000–V4000), both within each entire bulboclitoris structure and the portion not overlapping the PTV, reinforcing the value of SO‐VMAT in limiting high‐dose radiation to erectile tissue. The external genitalia also showed significant reductions in mean and maximum doses, as well as in V1000 and V2000, despite higher dose levels being largely absent in both planning techniques. While the vaginal D50% and mean dose did not differ significantly between the two techniques, the V4000 was significantly lower with SO‐VMAT. Collectively, these findings suggest that SO‐VMAT achieves superior sparing of critical female sexual structures.

Given that the vagina is often largely within the target volume and that the bulboclitoris is typically in close proximity, combining this SO‐VMAT approach with other organ sparing techniques, such as the use of a vaginal dilator during treatment to displace the anterior vaginal wall, bulboclitoris, and other important sexual structures further from the targeted area, may maximize the potential for sexual organ sparing in these patients.^25^, ^26^, ^27^, ^28^ Establishing techniques to reduce dose to these structures both with or without a dilator in place during treatment remains important given that many patients are unable to tolerate the dilator or choose to proceed without a dilator in place during treatment; however, when feasible, dilator‐assisted simulation and treatments can provide additional opportunities for reproducible vaginal wall sparing.

In our institutional experience, dilator‐assisted simulation and treatment, when tolerated, can reproducibly delineate the vaginal wall and can reduce high‐dose exposure, particularly to the anterior portion most proximate to the PTV. Recent reports in anorectal cancer patients have likewise shown that dilator use during pelvic radiotherapy reduces anterior vaginal wall dose and improves patient‐reported outcomes, with proposed constraints including keeping AVW V35 Gy below 60% and recognizing AVW D50% above 48 Gy as a predictor of sexual dysfunction.^25^, ^26^, ^27^ Beyond these dosimetric advantages, vaginal dilators represent a practical, low‐cost intervention that can be readily integrated into clinical workflows, and future studies combining their use with SO‐IMRT may help establish optimized protocols for sexual‐function–preserving radiotherapy.

Among the evaluated nonsexual structures, SO‐VMAT also significantly reduced the percent volume of the bladder receiving at least 5000 cGy, while doses to the bowels, pelvic bones, and femoral heads were not significantly different. These findings suggest that compared to standard 3D‐CRT, SO‐VMAT may provide effective and safe dose sparing for sexual OARs and the bladder without compromising PTV coverage or increasing doses to other critical structures. However, it is important to acknowledge that when doses are reduced to certain structures, a dose redistribution may occur. While no significant differences were observed for most non‐sexual OARs, femoral head V3000cGy values were approximately 200%–300% higher with SO‐VMAT, increasing from 2.7%–2.9% to 11.3%–13.9% and maximum doses remained under 4500cGy but with a 5.5%–5.7% increase. While these values remain well below institutional and commonly employed dose constraints (V3000cGy < 50% and Dmax < 4500cGy–5000cGy) in both groups, it is important to evaluate the safety impacts of these tradeoffs. Small increases in low dose spread to surrounding tissues, such as the skin, were also observed. These findings reflect expected tradeoffs in VMAT planning and underscore the importance of balancing dose‐sparing priorities across all critical structures.

Beyond reductions in OAR doses, the IDL analysis confirmed that SO‐VMAT achieved improved high‐ and intermediate‐dose conformality compared to 3D‐CRT, as evidenced by significant reductions in the 100% and 50% IDL volumes. Although the 105% isodose volume was not significantly different between techniques, SO‐VMAT consistently reduced the spread of high‐dose regions while slightly increasing low‐dose exposure at the 5% isodose level, a pattern consistent with prior observations in VMAT. A similar trend was observed for dose to the skin surface, where SO‐VMAT significantly reduced the volume receiving more than 2000 cGy but increased low‐dose exposure at 500 cGy. These findings reinforce the potential for SO‐VMAT to concentrate therapeutic doses within the target while minimizing hotspots and redistributing dose away from the prioritized adjacent normal sexual tissues if the tradeoff is deemed acceptable.

Given the current absence of robust data on radiation‐induced sexual dysfunction related to erectile tissue function in female patients, extrapolation from studies in prostate cancer may provide useful insights. For example, Sethi et al. found that IMRT allowed for dose escalation in prostate cancer while dose sparing of penile erectile tissues compared to 3D‐CRT.^29^ However, like our study, clinical or quality‐of‐life outcomes were not assessed. Data for sparing the function of the erectile tissues via sparing of erectile and neurovascular structures are mixed. Suggested erectile tissue constraints include a D2% of less than 50Gy and a mean dose of less than 2000cGy EQD2.^30^ By comparison, SO‐VMAT was capable of reducing mean bulboclitoris dose from 2206.9 cGy with 3D‐CRT to 1751.3 cGy, suggesting a clinically meaningful benefit. Nevertheless, collection of detailed functional outcomes for female erectile tissues and dose‐volume response modeling are needed to understand the unique tissue tolerance of the bulboclitoris as well as the supplying neurovasculature in order to quantify the clinical benefit of SO‐VMAT.

Our study is among the first to investigate the effects of different radiotherapy techniques in a patient cohort with low rectal cancer on dosimetric indices for female sexual OARs, particularly the bulboclitoris and vagina. Previous studies in anal cancer have demonstrated that IMRT techniques reduce radiation doses to the external genitalia and bladder, consistent with our findings.^31^, ^32^ The RTOG 0529 trial showed that IMRT improved hematologic, gastrointestinal, and dermatologic toxicity compared to 3D‐CRT, leading to its adoption as the standard of care for anal cancer.^33^, ^34^ For rectal cancer, studies comparing IMRT and 3D‐CRT have primarily focused on nonsexual OARs. For instance, Mok et al. found that IMRT reduced radiation doses to the bladder and small bowel but did not investigate that to the sexual OARs.^35^ A meta‐analysis further supported IMRT's ability to reduce acute gastrointestinal and genitourinary toxicities, yet none of the included studies evaluated sexual structures or sexual toxicities.^36^ Moreover, the RTOG 0822 phase II trial found no significant reduction in gastrointestinal toxicities with IMRT, which has limited its adoption as a standard of care in rectal cancer.^19^ Importantly, a recent secondary analysis of the RAPIDO trial reported that IMRT was associated with higher rates of late sexual toxicity compared with 3D‐CRT, despite no differences in GI or GU outcomes, likely reflecting that IMRT was delivered without specific sexual organ–sparing.^20^ Taken together, these gaps highlight the importance of our study in demonstrating that SO‐VMAT, rather than VMAT alone, may be necessary to reduce radiation dose to female sexual OARs and improve long‐term outcomes for women with rectal cancer.

Despite its strengths, our study has several limitations. Most notably, the small sample size limits the generalizability of our findings and reduces statistical power, even with the use of nonparametric methods. This may have prevented some true differences from reaching statistical significance. While this limited cohort size reduces statistical power and prevents broader conclusions about population‐level outcomes, several comparisons still reached statistical significance, underscoring the consistency and magnitude of the observed differences. Accordingly, this study should be viewed as a proof‐of‐concept analysis demonstrating the feasibility of contouring female sexual OARs and providing preliminary dose–volume benchmarks for future studies.

Additionally, the absence of clinical and patient‐reported outcomes limits our ability to directly correlate dosimetric improvements with sexual function or quality of life. The retrospective nature of the study also introduces potential for selection bias. Nonetheless, our findings demonstrate consistent and meaningful dose reductions with SO‐VMAT, particularly in volumetric parameters, suggesting a promising advantage that warrants further investigation. Prospective studies with larger cohorts and integrated patient‐reported outcomes are needed to validate these results and support future changes in clinical practice.

Future studies with larger, multi‐institutional cohorts and integration of patient‐reported outcomes will be essential to validate these results, improve statistical robustness, and help define dose‐volume parameters that may guide treatment planning in clinical practice. Our findings provide early evidence supporting the development of preliminary DVH constraints for female sexual OARs, laying a foundation for future studies to evaluate dose–response relationships and inform treatment planning. Ultimately, incorporation of these structures into consensus contouring atlases and clinical guidelines will be critical to advancing sexual‐function–preserving RT for pelvic cancers. Together, these findings support the integration of sexual organ‐sparing strategies like SO‐VMAT into rectal cancer RT to optimize long‐term quality of life outcomes for female patients.

## 5 CONCLUSION

This feasibility study demonstrates that female sexual OARs can be systematically contoured in rectal cancer radiotherapy and that SO‐VMAT can achieve meaningful dose reductions to these structures compared with 3D‐CRT, without compromising target coverage. These findings highlight the importance of intentional sexual organ contouring, as VMAT delivered without specific sparing may not reduce, and may even increase sexual toxicity compared with 3D‐CRT. Preliminary dose–volume data from this study can inform future sexual organ–sparing strategies, but larger studies with patient‐reported outcomes are needed to validate these observations and define clinically relevant constraints.

## AUTHOR CONTRIBUTIONS

Margaret H. Downes contributed to study conception and design, data analysis, interpretation of results, and manuscript drafting. Victoria Olsen, Rendi Sheu, and Vishruta Dumane performed treatment planning and dosimetric data extraction. Andre Williams reviewed and verified all anatomical contours. Maria Thor provided methodological guidance and manuscript revision. Lucy Greenwald contributed to clinical interpretation and critical revision of the manuscript. Kristin Hsieh and Ayesha Ali provided clinical expertise in radiation oncology and manuscript editing. Orly Morgan assisted with data organization and literature review. Michael Buckstein treated all patients included in the study, contributed to study supervision, and critically revised the manuscript. Deborah C. Marshall supervised the overall project, contributed majorly to study conception and design, and critically revised the manuscript for intellectual content. All authors reviewed and approved the final manuscript.

## CONFLICT OF INTEREST STATEMENT

The authors declare no conflicts of interest.
